# FAT10 Induces cancer cell migration by stabilizing phosphorylated ABI3/NESH

**DOI:** 10.1080/19768354.2023.2186486

**Published:** 2023-03-11

**Authors:** Hyojin Um, Hoim Jeong, Beomgu Lee, Yerin Kim, Jihyeon Lee, Jong Seong Roh, Seung-Geun Lee, Hae Ryoun Park, William H. Robinson, Dong Hyun Sohn

**Affiliations:** a Department of Microbiology and Immunology, Pusan National University School of Medicine, Yangsan, Republic of Korea; b Department of Herbal Prescription, College of Korean Medicine, Daegu Haany University, Gyeongsan, Republic of Korea; c Biomedical Research Institute, Pusan National University Hospital, Busan, Republic of Korea; d Division of Rheumatology, Department of Internal Medicine, Pusan National University School of Medicine, Pusan National University Hospital, Busan, Republic of Korea; e Department of Oral Pathology, School of Dentistry, Pusan National University, Yangsan, Republic of Korea; f Division of Immunology and Rheumatology, Department of Medicine, Stanford University School of Medicine, Stanford, CA, USA; g VA Palo Alto Health Care System, Palo Alto, CA, USA

**Keywords:** ABI family member 3 (ABI3)/new molecule including SH3 (NESH), WAVE regulatory complex (WRC), human leukocyte antigen-F adjacent transcript 10 (FAT10), phosphorylation

## Abstract

The WAVE regulatory complex (WRC) is involved in various cellular processes by regulating actin polymerization. The dysregulation of WRC components is associated with cancer development. ABI family member 3 (ABI3)/new molecule including SH3 (NESH) is one of the WRC components and it has been reported that ABI3 phosphorylation can affect WRC function. Although several residues of ABI3 have been reported to be possible phosphorylation sites, it is still unclear which residues are important for the function of ABI3. Furthermore, it is unclear how the phosphorylated form of ABI3 is regulated. Here, we demonstrate that ABI3 is stabilized by its interaction with human leukocyte antigen-F adjacent transcript 10 (FAT10). Using phospho-dead or phospho-mimetic mutants of ABI3, we showed that serine 213 and 216 are important phosphorylation sites of ABI3. In particular, FAT10 has a higher affinity for the phosphorylated form of ABI3 than the non-phosphorylated form, and it stabilizes the phosphorylated form more than the non-phosphorylated form through this differential affinity. The interaction between FAT10 and the phosphorylated form of ABI3 promoted cancer cell migration. Therefore, our results suggest that FAT10 stabilizes the phosphorylated form of ABI3, which may lead to WRC activation, thereby promoting cancer cell migration.

## Introduction

The WAVE regulatory complex (WRC) regulates polymerization of the actin cytoskeleton by stimulating the Arp2/3 complex. It consists of five subunits: HSPC300, ABI1 (ABI2 or ABI3), NAP1/HEM2 (HEM1), SRA1/CYFIP1 (PIR121/CYFIP2), and WAVE1 (WAVE2 or WAVE3) (Chen et al. 2014). Since WRC plays a key role in cellular processes, including the maintenance of cellular structure, spreading, adhesion, and migration, dysregulation of WRC components is known to be related to cancer development (Kurisu et al. 2005; Yamaguchi and Condeelis 2007; Silva et al. 2009; Escobar et al. 2010; Rana et al. 2021).

The ABI family member 3 (ABI3), also known as new molecule including SH3 (NESH), is one of ABL-interactor (ABI) family proteins which include ABI1 and ABI2 (Leng et al. 2005). It has been suggested that ABI3-containing WRC is functionally distinct from ABI1/2-based WRC. When ABI3 is ectopically expressed, WAVE2 translocation to the plasma membrane is significantly reduced, and the peripheral lamellipodial structure is disturbed (Sekino et al. 2015). Additionally, although ABI1 and ABI2 were able to phosphorylate WAVE2, ABI3 did not (Hirao et al. 2006).

It has been suggested that the phosphorylation of ABI3 by the PI3K/AKT pathway inhibits inactive WRC formation, thereby activating WRC (Moraes et al. 2017; Rana et al. 2021). Abnormalities in the PI3K pathway are common in cancers and play an important role in neoplastic transformation. Therefore, although ABI3 is considered a tumor suppressor, the phosphorylated form of ABI3 may be associated with cancer development by activating WRC. There are several putative phosphorylation sites in ABI3, including serine 213, 216, and 342, which are highly conserved among humans, mice, rats, cows, and monkeys (Moraes et al. 2017). Although it has been reported that these residues are likely to be phosphorylation sites, it remains unclear which residues of ABI3 are actually phosphorylated and important for the function of ABI3.

A growing number of proteins containing domains with significant homology to ubiquitin have been discovered over the past several years. Ubiquitin-like proteins (UBLs), such as SUMO, NEDD8, and ISG15 have been investigated in detail, and specific E1, E2, and E3 enzymes have been identified (Jentsch and Pyrowolakis 2000). Human leukocyte antigen-F adjacent transcript 10 (FAT10) is a fairly new member of the UBL family that covalently modifies target substrates by binding via the conserved di-glycine motif at the C-terminus (Fan et al. 1996). FAT10 is encoded in the major histocompatibility complex and is synergistically induced by the proinflammatory cytokines, interferon gamma (IFN-γ) and tumor necrosis factor-alpha (TNF-α) (Liu et al. 1999; Raasi et al. 1999). Additionally, FAT10 as well as its conjugates are rapidly degraded by the proteasome in a ubiquitin-independent manner (Hipp et al. 2005), indicating that FAT10 is the first ubiquitin-like modifier that functions as a protein signal for the rapid degradation of substrate proteins through the proteasome.

FAT10 has recently been highlighted for its association with cancer, and is overexpressed in various cancers, such as gastrointestinal cancer, liver cancer, pancreatic ductal adenocarcinoma, and glioma (Yuan et al. 2014). Additionally, FAT10 also suppresses the transcriptional activity of the tumor suppressor gene p53 and facilitates p53 degradation to accelerate tumorigenesis (Choi et al. 2014; Su et al. 2021; Zhang et al. 2021). Furthermore, overexpression of FAT10 promotes tumor growth (Theng et al. 2014) and malignancy, suggesting that FAT10 may play a key role in tumor development. In this study, we hypothesized that FAT10 may regulate ABI3 through interaction, which contributes to cancer development. We demonstrated that FAT10 induced cancer cell migration by preferentially stabilizing phosphorylated ABI3.

## Materials and methods

### Cell culture and reagents

Human embryonic kidney (HEK)−293T cells were cultured in the Dulbecco's modified eagle medium (Sigma-Aldrich) supplemented with 10% fetal bovine serum (FBS), 100 units/mL of penicillin, and 100 μg/mL of streptomycin (Thermo Fisher Scientific). Human colorectal adenocarcinoma SW480 cells were cultured in the Roswell Park Memorial Institute-1640 medium (Sigma-Aldrich) supplemented with 10% FBS, 100 units/mL of penicillin, and 100 μg/mL of streptomycin. Cells were cultured in a humidified atmosphere containing 5% CO₂ at 37°C. Lipofectamine 3000 transfection reagent was purchased from Thermo Fisher Scientific. GENE-fect was purchased from TransLab. Cycloheximide was purchased from Sigma-Aldrich. Protease and phosphatase inhibitor cocktails were purchased from GenDEPOT. The IP lysis buffer and Dynabeads protein G were purchased from Thermo Fisher Scientific. The anti-Myc (9E10)-HRP antibody was purchased from Abcam, and the anti-FLAG (M2)-HRP, anti-HA-HRP (3F10), anti-FLAG-M2, anti-Myc (9E10), and β-actin-HRP antibodies were purchased from Sigma-Aldrich.

### Plasmids

The cDNA for ABI3 was purchased from the Korean Human Gene Bank, and the cDNA for FAT10 was purchased from Origene. p3xFLAG-CMV10 or pcDNA3.1-Myc-His C was used to express FLAG-FAT10 and ABI3-Myc, respectively. The sequences of all plasmids were verified by sequencing (Bionics). Plasmids for transfection were extracted using a Midi Prep Kit (Qiagen).

### ABI3 site-directed mutagenesis

Information on the putative phosphorylation sites of human ABI3 was obtained from the NCBI (https://www.ncbi.nlm.nih.gov/nuccore/NM_016428.3) and UniProt (https://www.uniprot.org/uniprotkb/Q9P2A4/entry). Residue numbers were defined according to human isoform 1 (NP_057512.2), with S213, S216, and S342 as putative phosphorylation sites. Substitution of serine with alanine or aspartic acid was used for the production of phospho-dead mutant or phospho-mimetic mutants, respectively, as previously described (Lee et al. 2011). Site-directed mutagenesis was performed using primers for phospho-dead (S213A, S216A, and S342A) and phospho-mimetic (S213D, S216D, and S342D) mutations, and confirmed by sequencing. Wild-type and mutant ABI3 expression plasmids were transiently transfected into HEK-293 T cells, and the expression of mutant proteins was evaluated by western blot analysis.

### Transfection and preparation of protein lysates

HEK-293T cells were plated in a 6-well plate at a density of 0.7 × 10^6^ cells/well a day before transfection. Transfection of plasmids was performed using lipofectamine 3000 transfection reagent or GENE-fect, according to the manufacturer’s instructions. For some experiments, cycloheximide was used before cell harvest. Cells were harvested after washing with 1 × phosphate buffered saline (PBS) and lysed with IP lysis buffer supplemented with protease and phosphatase inhibitor cocktails. The lysates were centrifuged at 13,000 rpm for 15 min at 4°C, and the supernatant was used for immunoprecipitation or western blot analysis. Total protein concentration was determined using bicinchoninic acid (BCA) method (Thermo Fisher Scientific).

### Western blot analysis

For western blot analysis, cell lysates were resolved on 8% or 10% sodium dodecyl sulfate-polyacrylamide gel electrophoresis (SDS-PAGE) and transferred to polyvinylidene fluoride (PVDF) membranes (Merck Millipore). Subsequently, the membrane was dried at 37°C for 1 h, followed by blocking with 5% skim milk in 1 × Tris-buffered saline supplemented with 0.1% Tween-20 (TBS-T) for 1 h. The membrane was then probed for at least 2 h with anti-Myc (9E10)-HRP or anti-FLAG (M2)-HRP antibodies, followed by washing three times with TBS-T for 10 min. Finally, the membranes were visualized using an enhanced chemiluminescence reagent (ProNA ECL Ottimo, TransLab). Chemiluminescent signals were detected using an ImageQuant LAS3000 Analyzer (FujiFilm). The band intensities were quantified using ImageJ software. The data were normalized to β-actin expression (loading control) as previously described (Roh et al. 2021).

### Co-immunoprecipitation assay

Cells were harvested and lysed on ice for 15 min in the IP lysis buffer containing protease and phosphatase inhibitor cocktails, and the lysates were centrifuged at 13,000 rpm for 15 min at 4°C. For immunoprecipitation, 4 μg of anti-Myc (9E10) or anti-FLAG (M2) was added and incubated overnight at 4°C on a tube rotator. Dynabeads protein G was added and incubated for 1 h at 4°C on a tube rotator. The immunoprecipitate was washed four times with IP lysis buffer (0.5 mL). After the final washing, the beads were resuspended in 25 μL 2 × sample buffer. The samples were boiled for 5 min, resolved by SDS-PAGE, and western blot analysis was performed.

### Wound healing assay

Human colorectal adenocarcinoma SW480 cells were seeded in a 24-well plate and grown overnight until the density reached 80%. The monolayer was scratched using an SPLScar^TM^ Scratcher (SPL) and the floating cells were removed. The migration of SW480 cells to the scratched areas was assayed after incubation for 24 h and observed using an optical microscope CKX41 (Olympus). Migration rate was quantified using the ImageJ plugin, MRI wound healing tool.

### Statistical analysis

Statistical analyses were performed using the GraphPad Prism software. All data are presented as mean ± standard error of the mean (SEM). One-way analysis of variance (ANOVA) with Tukey’s multiple comparison test was used for comparisons between multiple groups, and *p* < 0.05 was considered statistically significant.

## Results

### ABI3 interacts with FAT10

**Figure 1.**
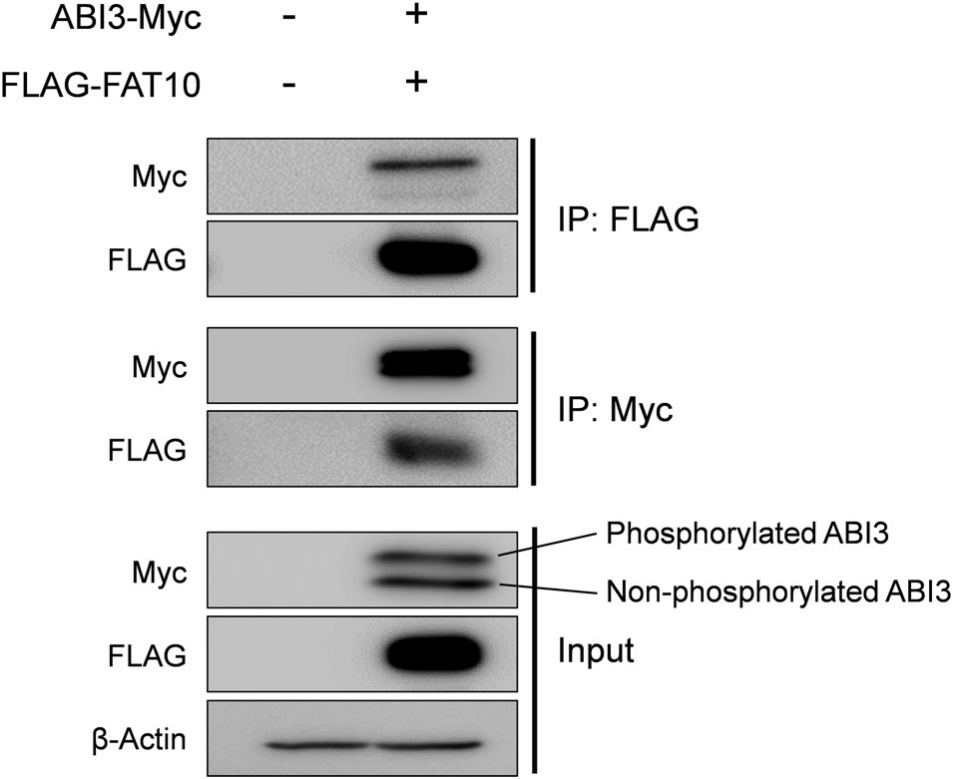
Interaction of ABI3 with FAT10. HEK-293T cells were co-transfected with Myc-tagged ABI3 (ABI3-Myc) and FLAG-tagged FAT10 (FLAG-FAT10) expression plasmids. Cell lysates (300 μg) were immunoprecipitated using anti-FLAG or anti-Myc antibodies. Western blot analysis was performed using anti-Myc-HRP or anti-FLAG-HRP. Beta-actin was used as the loading control. The upper band of ABI3-Myc is phosphorylated form and the lower band is non-phosphorylated form.

It has been reported that ABI3 expression is decreased in thyroid tumors, and ectopic expression of ABI3 inhibits tumor formation (Latini et al. 2011). In contrast, it has been suggested that phosphorylation of ABI3 may disrupt inactive WRC formation, which may lead to cancer development through the activation of WRC (Moraes et al. 2017; Rana et al. 2021). However, it is still unclear whether phosphorylation of ABI3 contributes to cancer development and how the phosphorylated form of ABI3 is regulated. In various cancers, FAT10 is upregulated and is known to regulate several proteins involved in cancer development, such as MAD2, p53, and β-catenin through covalent and non-covalent interactions (Aichem and Groettrup 2016; Su et al. 2021; Zhang et al. 2021). Therefore, we investigated whether ABI3 is regulated by FAT10. First, co-immunoprecipitation was performed to examine the interactions between ABI3 and FAT10. As shown in Figure 1, Myc-tagged ABI3 was co-immunoprecipitated with FLAG-tagged FAT10. Furthermore, considering the input control, which shows weaker band intensity of the phosphorylated form compared to the non-phosphorylated form of ABI3, the phosphorylated form interacts more strongly with FAT10 than the non-phosphorylated form. This result shows that the phosphorylated form of ABI3 may have a higher affinity for FAT10 than the non-phosphorylated form.

### Main phosphorylation sites on ABI3 are serine 213 and 216

**Figure 2.**
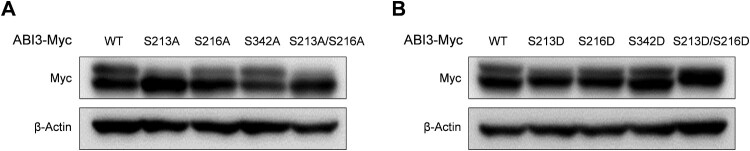
Main phosphorylation sites on ABI3 are S213 and S216. HEK-293 T cells were transfected with either phospho-dead mutants (A) or phospho-mimetic mutants of ABI3 (B), along with wild-type ABI3. The levels of the phosphorylated and non-phosphorylated forms of wild-type or mutant ABI3 were determined by western blot analysis using an anti-Myc-HRP antibody. Beta-actin was used as the loading control.

Based on previous studies and database searches, S213, S216, and S342 in ABI3 are potential phosphorylation sites. To identify the main phosphorylation sites in ABI3, we introduced mutations at each potential phosphorylation site. Phospho-dead mutants of ABI3 were produced by substituting serine with alanine (S213A, S216A, and S342A), and the phospho-mimetic mutants of ABI3 were produced by substituting serine with aspartic acid (S213D, S216D, and S342D). Western blot analysis indicated that while ABI3 (S342A) showed almost similar amount of phosphorylated form as the wild-type, ABI3 (S213A) and ABI3 (S216A) showed relatively low amounts of phosphorylated forms (Figure 2A). Moreover, while the phosphorylated form disappeared in ABI3 (S213A/S216A), non-phosphorylated form disappeared in ABI3 (S213D/S216D) (Figure 2). These results suggest that S213 and S216 on ABI3 are important phosphorylation sites.

### FAT10 has a higher affinity for phosphorylated form of ABI3 than non-phosphorylated form

**Figure 3.**
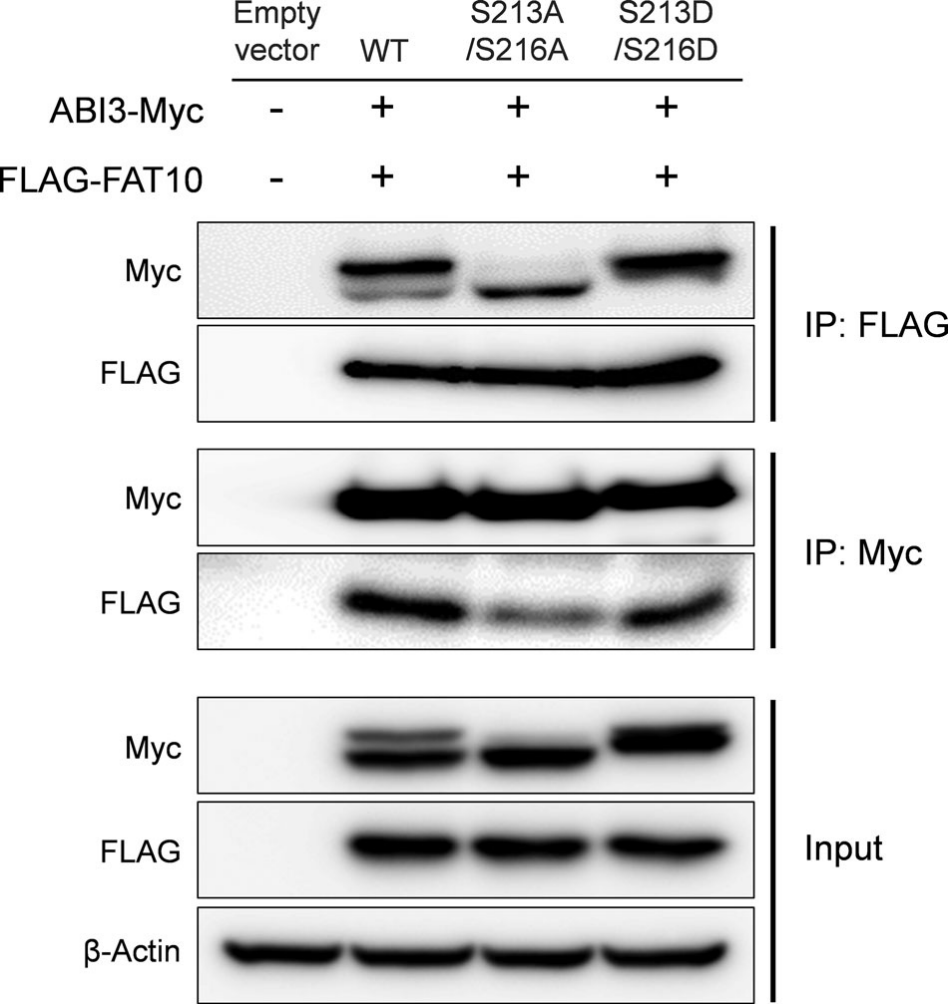
Phosphorylated form of ABI3 has a higher affinity for FAT10. HEK-293 T cells were co-transfected with ABI3-Myc, ABI3 (S213A/S216A)-Myc, or ABI3 (S213D/S216D)-Myc expression plasmids, along with the FLAG-FAT10 expression plasmid. Cell lysates (300 μg) were immunoprecipitated using anti-FLAG or anti-Myc antibodies. Western blot analysis was performed using anti-Myc-HRP or anti-FLAG-HRP antibodies. Beta-actin was used as the loading control.

Next, we tested whether the phospho-dead or phospho-mimetic mutants of ABI3 have different binding affinities for FAT10, as suggested in Figure 1. The expression plasmids for the phospho-dead mutant of ABI3 (S213A/S216A) or phospho-mimetic mutant of ABI3 (S213D/S216D) were co-transfected with the FLAG-FAT10 expression plasmid, and the interaction between ABI3 mutants and FAT10 was investigated by co-immunoprecipitation. As shown in Figure 3, the wild-type and its mutants interacted with FAT10. However, compared to the input control, the immunoprecipitate of the phospho-mimetic mutant showed a stronger signal than the phospho-dead mutant, which again confirms that the phosphorylated form of ABI3 has a higher binding affinity for FAT10 than the non-phosphorylated form.

### Phosphorylation status of ABI3 is important for the stabilization of ABI3 by FAT10

**Figure 4.**
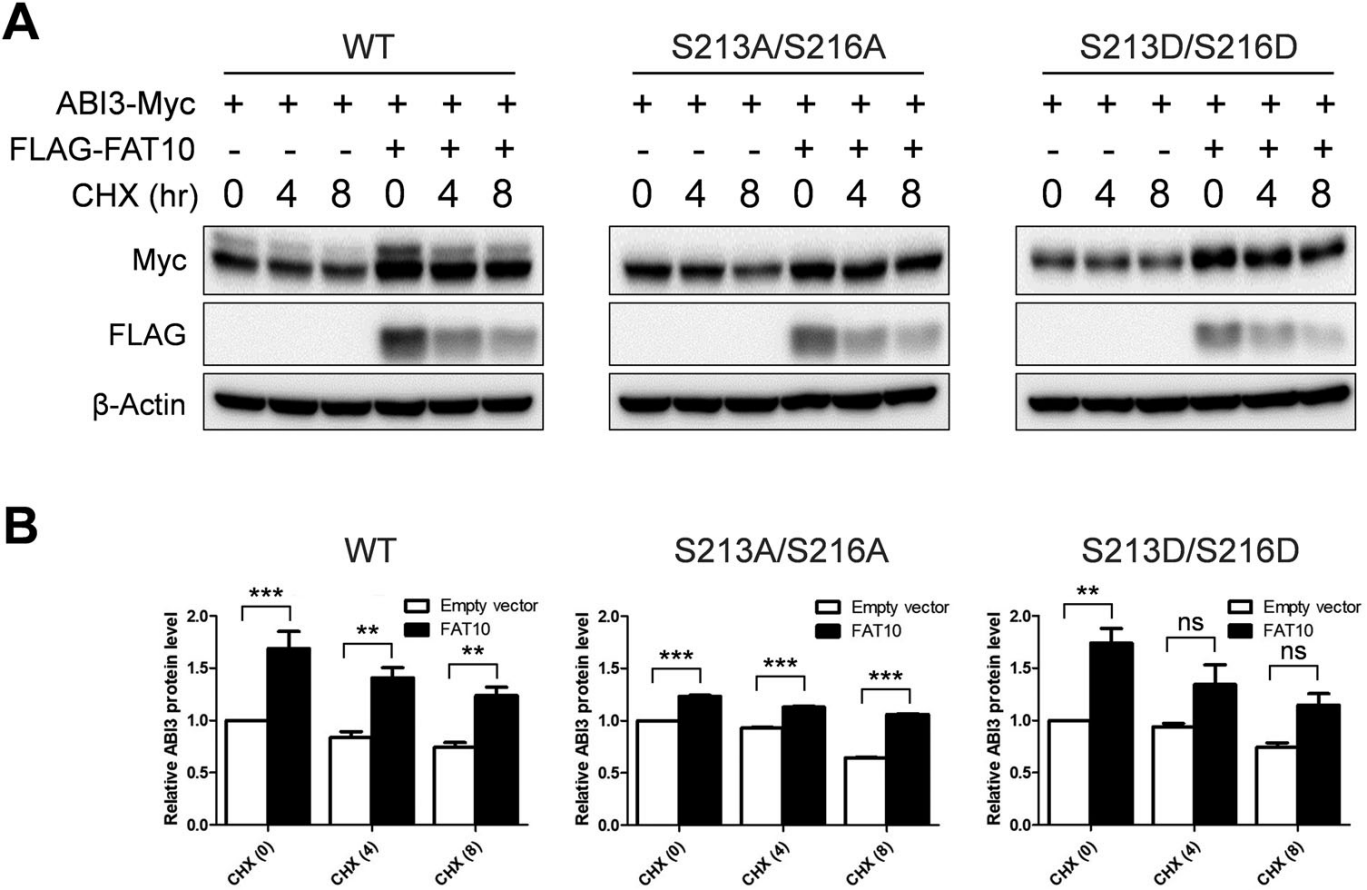
Phosphorylation status of ABI3 is important for the stabilization of ABI3 by FAT10. (A) HEK-293T cells were co-transfected with wild-type, phospho-dead, or phospho-mimetic mutants of ABI3-Myc expression plasmids, along with or without the FLAG-FAT10 expression plasmid. Cells were treated with cycloheximide (CHX, 100 μM) for 0, 4, 8 h. Western blot analysis was performed with anti-Myc-HRP or anti-FLAG-HRP antibodies. Beta-actin was used as the loading control. (B) Data from western blot were quantified by densitometry. Data are presented as mean ± SEM and *p* values were calculated by one-way ANOVA with Tukey’s multiple comparison test. **p < 0.01, ***p < 0.001. ns, not significant.

FAT10 targets its substrates for proteasomal degradation by covalent binding in a manner similar to that of ubiquitin. However, recent studies have demonstrated that FAT10 stabilizes its interacting proteins through non-covalent interactions (Yuan et al. 2014; Dong et al. 2016; Liu et al. 2016; Zhou et al. 2018; Zou et al. 2018). As shown in Figures 1 and 3, ABI3 non-covalently interacts with FAT10. Additionally, we did not detect any covalent interactions between ABI3 and FAT10 (data not shown). Therefore, it is possible that ABI3 is also stabilized by FAT10, and that the phosphorylation status of ABI3 may be important for stabilization. To verify this possibility, wild-type or phospho-mutants of the ABI3-Myc expression plasmid were transfected with or without the FLAG-FAT10 expression plasmid and a cycloheximide chase assay was performed. As expected, ABI3 was stabilized by FAT10, and the phospho-mimetic mutant of ABI3 showed a greater stabilization effect than the phospho-dead mutant (Figure 4). However, the degradation rate of each mutant was similar, even in the co-expression of FAT10, probably because of the very rapid degradation of FAT10 during the cycloheximide chase assay. These results suggest that ABI3 is stabilized by FAT10, and that phosphorylation of ABI3 is important for stabilization by FAT10.

### Phosphorylated ABI3-induced cancer cell migration is mediated by FAT10

**Figure 5.**
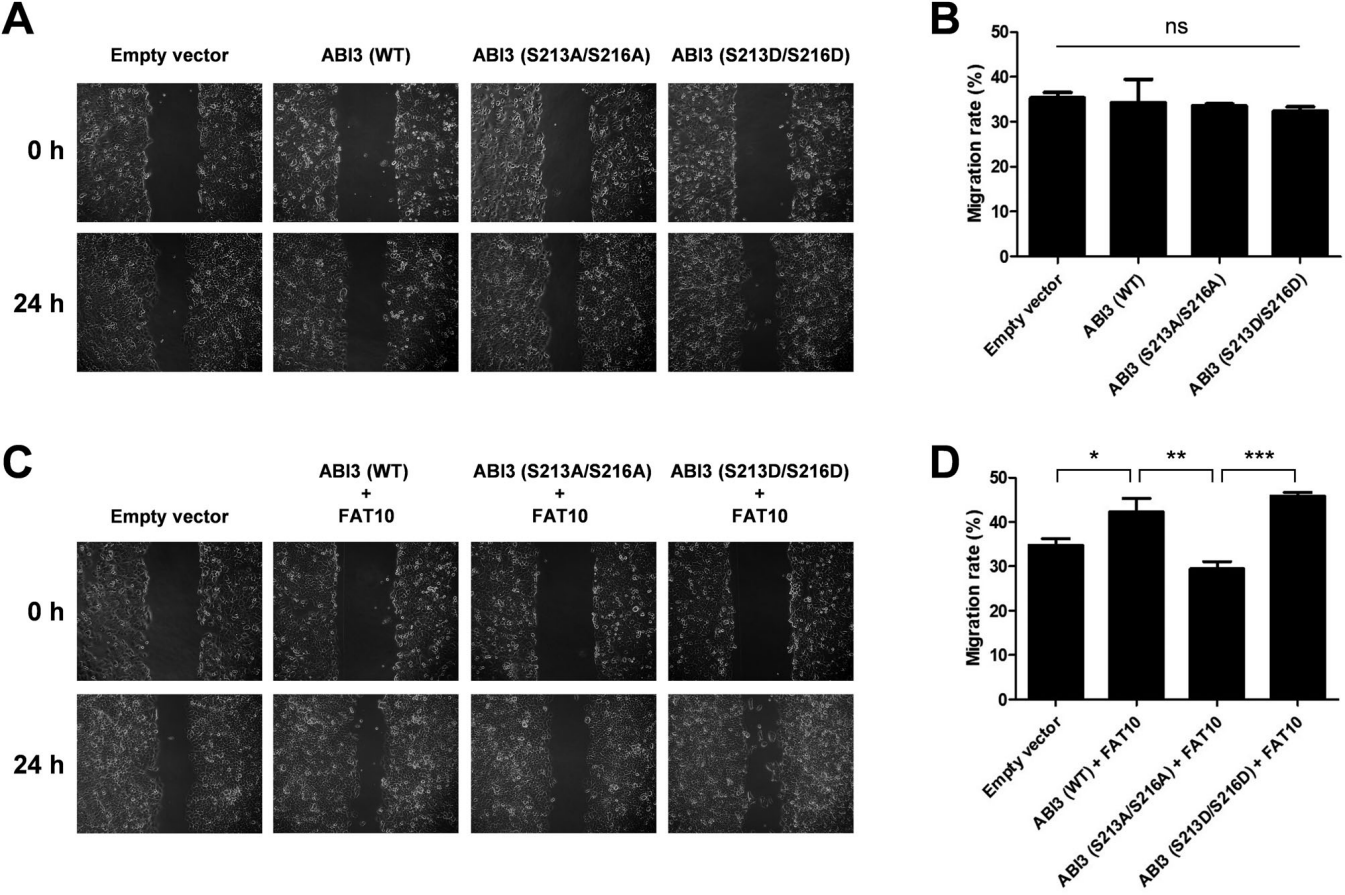
Phosphorylated ABI3-induced cancer cell migration is mediated by FAT10. (A and C) SW480 cells were transfected with wild-type, phospho-dead, or phospho-mimetic mutants of ABI3-Myc expression plasmids, along with or without the FLAG-FAT10 expression plasmid and the scratched areas of cells were observed at 0 and 24 h by an optical microscope (x100). (B and D) The migration rate was calculated by comparing the scratched area at 0 and 24 h. Data are presented as mean ± SEM and *p* values were calculated by one-way ANOVA with Tukey’s multiple comparison test. *p < 0.05, **p < 0.01, ***p < 0.001. ns, not significant.

Considering that FAT10 is overexpressed in various cancers and stabilizes phosphorylated ABI3 (Figure 4), it is possible that the interaction between FAT10 and phosphorylated ABI3 contributes to cancer development. Therefore, we tested whether FAT10 mediates phosphorylated ABI3-induced cancer cell migration using a wound-healing assay. Overexpression of wild-type, phospho-dead mutant, or phospho-mimetic mutant of ABI3 alone in SW480 colon cancer cells had no effect on migration (Figure 5A and B). However, when FAT10 was overexpressed with wild-type ABI3 or its mutants, the wild-type or phospho-mimetic mutant of ABI3 promoted cancer cell migration, which was inhibited by the phospho-dead mutant (Figure 5C and D). These results suggest that the interaction between FAT10 and phosphorylated ABI3 contributes to the regulation of cancer cell migration.

## Discussion

It has been reported that FAT10 is involved in cancer development; it is overexpressed in various cancers, including colon and liver cancers (Lee et al. 2003). Additionally, FAT10 promotes the survival, proliferation, migration, and invasion of cancer cells (Gao et al. 2014; Aichem and Groettrup 2016). In particular, FAT10 suppresses the transcriptional activity of p53 and facilitates p53 degradation to accelerate tumorigenesis (Choi et al. 2014; Su et al. 2021; Zhang et al. 2021). These results showed that FAT10 plays an important role in tumorigenesis. Although diverse UBLs, including FAT10, NEDD8, SUMO, and ISG15, are similar to ubiquitin, FAT10 is the only UBL that targets its substrates for proteasomal degradation. However, recent studies have revealed that FAT10 can also stabilize interacting proteins. Non-covalent binding of FAT10 to β-catenin stabilizes β-catenin by blocking its ubiquitination (Yuan et al. 2014). Additionally, caveolin-3, eEF1A1, survivin, and ZEB2 are stabilized by FAT10 (Dong et al. 2016; Liu et al. 2016; Zhou et al. 2018; Zou et al. 2018). In this study, we also showed that ABI3 is stabilized by FAT10 through non-covalent binding (Figures 1 and 4), but the mechanism of this stabilization remains unclarified.

Although ABI3 plays an important role as a tumor suppressor, the precise mechanism by which ABI3 exerts this function is still unknown. It has been suggested that the phosphorylation of ABI3 by the PI3K/AKT pathway may disrupt inactive WRC formation, which may contribute to tumorigenesis. We showed that FAT10 mediates phosphorylated ABI3-induced cancer cell migration, probably through preferential interaction of FAT10 with phosphorylated ABI3. Considering the importance of phosphorylation of ABI3, we also investigated the phosphorylation sites of ABI3 and found that serine 213 and 216 are important phosphorylation sites. This is contrary to a previous report suggesting that serine 342 of ABI3 is likely a phosphorylation site by AKT (Moraes et al. 2017). However, their S342A mutant was still phosphorylated, according to their data. In contrast, we showed that mutation of both serine 213 and 216 into alanine completely abrogated the phosphorylation of ABI3, and mutation of both serine 213 and 216 into aspartic acid mimics the phosphorylated form of ABI3.

In this study, we confirmed that the phosphorylated form and phospho-mimetic mutant of ABI3 have a strong affinity for FAT10 compared to the non-phosphorylated form or phospho-dead mutant of ABI3. Additionally, FAT10 exhibited a stabilization effect by non-covalent binding with ABI3. More importantly, the phospho-mimetic mutant of ABI3 showed a greater stabilization effect than the phospho-dead mutant (Figure 4), confirming that phosphorylation status of ABI3 is important for stabilization by FAT10. Further studies are required to determine the detailed stabilization mechanisms of phosphorylated ABI3 by FAT10.

In conclusion, we demonstrated that FAT10 interacts with ABI3 and stabilizes it with preference for the phosphorylated form of ABI3. Our study suggests a potential key role for FAT10 in the tumor-inducing function of phosphorylated ABI3. Further research is necessary to fully understand the interaction between phosphorylated ABI3 and FAT10 in the regulation of cancer progression.

## Author contributions

Conceptualization: HU, HJ, HRP, and DHS; Acquisition of data: HU, HJ, BL, YK, JL, and JSR; Analysis and interpretation of data: HU, HJ, YK, SGL, HRP, WHR, and DHS; Writing and editing of the manuscript: HU, HJ, BL, YK, JL, JSR, and DHS; Funding acquisition: DHS; Study supervision: DHS.
